# Osteonecrosis of the jaw (ONJ) in patients who receive Bone Targeting Agents (BTAs): the power of e-learning

**DOI:** 10.3332/ecancer.2018.ed77

**Published:** 2018-01-23

**Authors:** Ourania Nicolatou-Galitis, Cesar Migliorati

**Affiliations:** 1 Dental School, National and Kapodistrian University of Athens, Athens 115 27, Greece; 2 Department of Oral and Diagnostic Sciences, University of Florida College of Dentistry, Gainesville, FL 32610-0412, USA

**Keywords:** osteonecrosis of the jaw (ONJ), bisphosphonates, denosumab, prevention, early diagnosis, management, dental extraction, e-learning, ecancer

## Abstract

The definition, pathobiology and risk factors of ONJ in cancer patients who receive BTAs are discussed in the recent ecancer module for osteonecrosis of the jaw (http://ecancer.org/education/module/276-osteonecrosis-of-the-jaw.php). ONJ prevention, early diagnosis and management are presented. The critical question of the performance of dental extraction, during BTA therapy, as indicated with the recent studies, is supported. The importance of the collaboration between dental and oncology professionals and the patients is highlighted and can be achieved through appropriate education. The ecancer modules are valuable tools for successful e-learning in medical oncology education, including ONJ.

## Introduction

Bone Targeting Agents (BTAs), such as bisphosphonates and denosumab, can reduce the risk of skeletal-related adverse events and are widely used to protect the skeleton in patients with osteoporosis or bone metastases from various types of cancer [1, 2]. Osteonecrosis of the jaw (ONJ), a rare but potentially serious adverse event, has been associated with cumulative doses of BTAs and may result in BTAs interruption. More than 90% of cases of ONJ occur in cancer patients, who receive high doses of i.v. bisphopshonates or denosumab. Appropriate prevention measures can reduce the risk of ONJ, while early diagnosis can lead to successful management.

## ONJ and ecancer e-learning module

**Figure 1.**
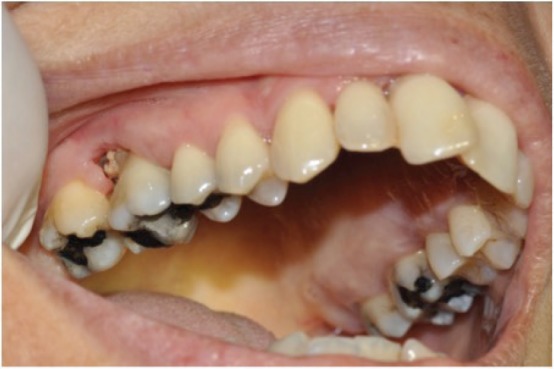
Exposed bone, right maxilla, periodontal area, associated with pain and tooth mobility, in a cancer patient with bone metastasis, who received BTA. No dental extraction had preceded the development of ONJ. *Courtesy of Dr Ourania Nicolatou-Galitis National & Kapodistrian University of Athens, Greece*.

Since the first reports of ONJ, in 2003, an increasing number of research data has been published and dedicated researchers continuously produce new knowledge. Osteonecrosis is classically an area of painful exposed necrotic bone on the mandible or maxilla. Today, we know that ONJ may also present as non-exposed bone [3, 4]. Cases of ONJ associated with anticancer drugs, which include classical chemotherapy agents, inhibitors of angiogenesis, tyrosine kinase and mammalian target of rapamycin inhibitors and immunotherapy agents have been reported [5]. The pathogenesis of ONJ in most likely multifactorial and infection seems to play an important role. Critical questions on ONJ have been clarified, while recently described modified surgical protocols seem to reduce the risk of ONJ following dental extraction [6, 7]. Only 3 among 89 patients with cancer receiving antiresorptives developed osteonecrosis following dental extractions, which were performed with alveolectomy and primary surgical closure [6]. Localized dental and periodontal disease, with histological necrosis of the alveolar bone may precede the clinical appearance of necrotic bone [8, 9]. This knowledge has re-enhanced our ONJ prevention protocols for oral health maintenance and has changed the globally accepted previous instruction for the avoidance of dental extraction in patients, who receive BTAs. ONJ may develop prior to dental extraction, while dental and/or jaw pain, swelling and tooth mobility are associated signs and symptoms [Figure 1]. As it is obvious, early ONJ, prior to dental extraction, may mimic localized dental or/and periodontal disease and infection and thus, it may be difficult to diagnose. Clinicians need adequate knowledge, obtained with education, in order to have a high suspicion index for ONJ early diagnosis. Early diagnosis of ONJ will lead to an effective management.

We also increasingly realize that the collaboration between dental and oncology professionals and patients is the key factor in the prevention and management of ONJ related to the bone targeted medications. Research and daily clinical practice have shown, beyond any doubt, that “education” can improve this multidisciplinary, successful collaboration.

The ecancer online learning ONJ module offers a unique tool for oncology training and education, always at a real time setting. Basic Knowledge on the mechanism of action of the bisphosphonates and denosumab, the updated clinical presentation of ONJ and the most recently described early diagnostic clinical criteria, the prevention, risk factors and effective management of ONJ are presented, through a successful multidisciplinary collaboration. Ecancer educational module on ONJ offers the appropriate knowledge to achieve ONJ prevention, early diagnosis and successful management, allowing for a successful BTAs protection of the bones in osteoporosis and in the cancer setting.

*E*cancer online educational module for ONJ was based on a program which evaluated the potential of online communities in knowledge measuring [8]. To our knowledge there are no other tools for online ONJ education and this was one of the reasons why the module was created. Online learning can be accessed anywhere at any time. We can keep up with the increasing oncology information within a safe setting. No delays in knowledge dissemination and education!

## Conclusion

Bone targeting agents, administered in osteoporosis or cancer with bone metastases, protect the skeleton and reduce skeletal-related adverse events, maintaining quality of life. BTAs have been associated with ONJ, a rare, but dose-limiting bone complication.

The ecancer online educational module offers information to clinicians prescribing these drugs. Their collaboration with dental professionals can contribute to improved and efficient prevention, early diagnosis and successful management of ONJ.

## Conflicts of interest

Ourania Nicolatou-Galitis has received consultant fees from AMGEN.

## Authors’ contributions

Ourania Nicolatou-Galitis wrote the article and Dr Cesar Migliorati edited it.
